# Acute Effects of ACL Injury-Prevention Warm-Up and Soccer-Specific Fatigue Protocol on Dynamic Knee Valgus in Youth Male Soccer Players

**DOI:** 10.3390/ijerph17155608

**Published:** 2020-08-04

**Authors:** Marco Andrés García-Luna, Juan Manuel Cortell-Tormo, Miguel García-Jaén, Manuel Ortega-Navarro, Juan Tortosa-Martínez

**Affiliations:** Department of General and Specific Didactics, Faculty of Education, University of Alicante, 03690 Alicante, Spain; marco.garcia.luna@gmail.com (M.A.G.-L.); m.garciajaen@ua.es (M.G.-J.); manuortegaft@gmail.com (M.O.-N.); juan.tortosa@ua.es (J.T.-M.)

**Keywords:** anterior cruciate ligament, football, sport, health, stability, neuromuscular control

## Abstract

Childhood anterior cruciate ligament (ACL) injuries—which can pose a major risk to a child’s sporting career—have been on the rise in the last few decades. Dynamic knee valgus (DKV) has been linked to an increased risk of ACL injury. Therefore, the aim of this study was to analyze the acute effects of an ACL injury prevention protocol (ACL-IPP) and a soccer-specific fatigue protocol (SSFP) on DKV in youth male soccer players. The research hypothesis was that DKV would be reduced by the ACL-IPP and increased by the SSFP. Eighteen youth male soccer players were divided according to baseline DKV. Those with moderate or large DKV performed a neuromuscular training protocol based on activation of the abductor and external rotator hip muscles. Those with little or no DKV performed a soccer-specific fatigue protocol. DKV was assessed using the single-leg squat pre- and post-protocols in both legs. The ACL-IPP significantly decreased DKV during single-leg squat (*p* < 0.01, effect size = 1.39), while the SSFP significantly increased baseline DKV in the dominant leg during single-leg squat (*p* = 0.012; effect size = 1.74). In conclusion, the ACL-IPP appears to acutely reduce the DKV in youth male soccer players, and the SSFP seems to acutely increase the DKV in those players who showed a light or no DKV in a non-fatigue situation. By using the SSFP, it may be possible to determine which players would benefit from injury prevention programs due to increased DKV during game scenarios, while hip abductor and external rotator neuromuscular training may be beneficial for players who have moderate and severe DKV during single-leg squat under non-fatigued scenarios.

## 1. Introduction

Jumping is one of the most common actions in sports. The vast majority of sports practices require jumps and explosive movements in the execution of their main sporting gestures. Thus, these skills can be considered as performance factors [1]. However, the landing pattern seems to influence to a great extent the forces received by the joints involved, especially the vertical forces [2] and therefore the risk of injury [3]. The type of injury in each sport varies, although particularly in soccer, the lower body is by far the most affected in all age ranges and performance levels [4,5]. The knee and the ankle appear to be the areas with the highest prevalence of injury in this sport [6] and nearly one-third of these injuries have been reported to be due to poor knee function [7]. In fact, between a third and a quarter of the soccer injuries occur without contact [7,8], which is quite worrying.

The anterior cruciate ligament (ACL) rupture is one of the most severe and prevalent injuries in soccer and ball sports [9], occurring mostly in noncontact situations [10,11]. Furthermore, ACL rupture in soccer becomes even more important, as it seems to be one of the most complex injuries to treat and the one which disables the athlete the longest [12,13,14]. In addition, even after a proper ACL reconstruction and rehabilitation, individuals often have impaired strength, proprioception, stability, balance and neuromuscular control [15], as well as an increased risk for ACL re-injury [16]. Aside from this increased re-injury risk, 59–70% of injured soccer players appear to develop knee osteoarthritis, with total knee arthroplasty required in 15% of those cases [17,18]. In fact, many of the injured players are not able to return to their pre-injury level of performance [19], which is extremely relevant.

Therefore, it is obvious that the ACL injury affects not only the performance or the quality of life of those involved, but also the economic burden on health systems, with estimated costs of around US$26 billion per year in the United States, including the treatments dedicated to reconstruction and rehabilitation [18,20]. Furthermore, it should also be noted that the number of ACL injuries in children and adolescents has increased considerably in the last years [21,22]. Due to the musculoskeletal immaturity of this population it seems that even more attention should be paid than in adults, since an injury at such a young age could have unexpected complications and even drastically limit the child’s future sports career [23].

Multiple theories regarding ACL injury (e.g., quadriceps shear force, axial loading or knee hyperextension) have been proposed in previous literature, although it is currently stated that the main mechanism of injury involves more than one plane of movement [24]. Thus, different studies have showed that knee valgus and the tibial rotation could be the main causes of ACL injury [24,25]. They are caused mainly in landings or abrupt changes of direction, in which the reaction forces with the ground may be five to seven times the body weight [26]. Dynamic knee valgus (DKV) is a modified pattern of movement or alteration in the alignment of the lower limb, mainly observed in the frontal plane [27] and with knee abduction load predicting 70–80% of ACL injury risk [3]. It should be noted that the occurrence of DKV is more pronounced in the female gender [28], although this does not mean that there is no risk in the male population [29]. Several factors have been analyzed as triggers of this alteration in knee movement, but two of the recent factors that have shown some evidence have been a reduced ankle dorsiflexion [30] and a deficit of strength or impaired activation of the abductor and adductor hip muscles, in particular a weakness in the abductors and external rotators of the hip [31,32]. Recent evidence suggests that knee and ankle bracing may reduce DKV [33,34].

The literature has demonstrated certain benefits and a reduced risk of ACL injury using ACL injury prevention programs [35]. Specifically, programs focusing on neuromuscular and proprioceptive enhancement have been shown to reduce the risk of ACL injury by 51–88% [36,37,38]. However, to the best of our knowledge, all preventive training programs proposed in the existing literature have been based on treatments lasting from several weeks to an entire season. Specific warm-up exercises have been shown to be effective in tolerating greater demands or requirements in sports practice and reducing the risk of injury [39]. Indeed, strengthening the hip abductor muscles has been proposed in several ACL injury prevention programs [40,41], although always in conjunction with other exercises and never in isolation. A recent study has shown that weakness of the hip abductor musculature (e.g., gluteus medius) predicts knee abduction moment and thus the risk of ACL injury [42]. Therefore, we hypothesized that a specific neuromuscular training of the hip abductor muscles during the warm-up would be capable to acutely decrease the knee abduction and the DKV during sports practice. This would be of great practical relevance in terms of reducing the risk of injury in the short term, without prejudice to the absolute importance of continuing to carry out, simultaneously, a long-term injury prevention program.

It is also widely recognized that most injuries, not only those related to the ACL, occur in the final stages of sports performance, which coincides with the presence of muscle fatigue [43]. Since muscles contribute to joint stability, neuromuscular fatigue has also been proposed as another risk factor for non-contact ACL injuries [44,45,46]. However, a recent review has concluded that the fatigue protocols published in the literature do not uniformly alter lower extremity biomechanical factors, due in part to the heterogeneity of the protocols and tasks proposed and suggests further research in this regard [47]. In addition, the few studies that have analyzed the effect of fatigue on DKV in pre-pubertal male children have used a bipodal drop–jump task as assessment method [48,49], while some studies have shown that one-leg tasks (e.g., such as a single leg squat) may be more useful in discriminating DKV because it requires greater stability and neuromuscular control [50,51]. Therefore, the objective of this study was to analyze the acute effects of an ACL injury prevention protocol (ACL-IPP) and a soccer-specific fatigue protocol (SSFP) on DKV in youth male soccer players. The research hypothesis was that DKV would be reduced by the ACL-IPP and increased by the SSFP.

## 2. Materials and Methods

### 2.1. Participants

A convenient sample of 18 youth male soccer players (age: 12.51 ± 0.87 years; weight: 48.72 ± 9.71 kg; height: 159.34 ± 9.74 cm; BMI: 19.12 ± 2.30 kg/m^2^), from categories U11 and U13, was recruited for this study. All had at least 6 years of training experience in amateur competitive level, training 3–4 days per week. To be included in the study, participants should have not suffered musculoskeletal injuries in the last six months. Parents or guardians signed an informed consent form detailing the purpose of the study and the protocols and procedures to be used. All the procedures were in accordance with the Declaration of Helsinki (ethical approval number: UA-2018-11-15, Research Ethics Committee of the University of Alicante).

### 2.2. Procedures

Before the pre-intervention evaluations, a standardized and guided warm-up was performed, consisting of joint mobility, light continuous running and dynamic stretching. Evaluations were conducted on an individual basis. The frontal plane of the single-leg squat (SLS) test on both legs—dominant and non-dominant—was recorded at different times during the intervention, with a high-definition camera with 4 K recording technology. The camera was placed 3 m away from the athlete and at the height of the subject’s knee above the ground, using a tripod. Prior to the recordings, three anatomic landmarks were bilaterally identified on athlete’s lower limbs with 2-cm-diameter markers. Afterwards, the videos were analyzed by two specialists with the 2D motion analysis software Kinovea v.0.8.15 (Kinovea open source project under GPLv2), which has demonstrated its validity and reliability in the literature for measuring angles and distances [52].

First, an ACL injury prevention protocol (ACL-IPP) with elastic bands was performed, focusing on neuromuscular and proprioceptive function of the gluteus medius. Five minutes before and after the protocol, the performance of the SLS test was recorded to analyze the pre–post-ACL-IPP differences. Second, and on a different day, participants who did not show DKV performing the SLS test participated in a soccer-specific fatigue protocol (SSFP), expressly designed for the study. Before the fatigue protocol and after reaching a fatigue level between 9–10 in the CR 0–10 scale [53], they performed the SLS, which was recorded to analyze the pre–post-SSFP differences.

#### 2.2.1. Single-Leg Squat (SLS) Test

The SLS was the chosen method for evaluation, as some authors have suggested that one-leg methods are better than two-leg ones at discriminating DKV [50,51]. The evaluation of each leg consisted of 3 trials, obtaining the average of the three with a variation coefficient of less than 10% [54]. Intraclass correlation coefficients (with 95% confidence limits) were calculated for each observer, and these demonstrated high and excellent values of relative reliability (0.902, 0.896 and 0.857, for DKV basal values, post-ACL-IPP and post-SSFP, respectively). Markers were placed in the anatomic areas of interest (i.e., anterior superior iliac spine; the midpoint of the tibiofemoral joint, on the patella; and the talocrural joint, on the frontal ankle area, at the level of the malleolus) for subsequent video analysis. The frontal-plane projection angle of the knee valgus was defined by the angle formed from a linear line that connects the anterior superior iliac spine with the midpoint of the tibiofemoral joint and a second line connecting the midpoint of the tibiofemoral joint and the talocrural joint. The maximum degree of DKV was evaluated, analyzing the maximum tibiofemoral angulation (frontal plane) in relation to the Q-angle, which is defined as the angulation in the anatomic reference position [55]. The difference (δ) between these two variables was used as the dynamic value of each participant, measured in degrees [31]. To stratify the sample according to the level of DKV presented in the basal situation, the total angulations were divided into three proportional ranges. Thus, participants were classified according to the following criteria: null or light DKV (0° ≤ δ ≤ 16.2°); moderate DKV (16.3° ≤ δ ≤ 32.4°); severe DKV (δ ≥ 32.5°).

Prior to the SLS test, the participants performed a bilateral knee flexion from the standing position until they reached 60° of knee flexion, measured by a digital goniometer (Digital Baseline Absolute + Axis Goniometer, Model 12–1027, version 7–08, Fabrication Enterprises, Inc., White Plains, New York, NY, USA). In that position, a string was placed in contact with the knee, which was the reference depth that the participant had to reach in the SLS test. From a one-leg standing position with arms crossed on the chest, the participant was instructed to perform the SLS, doing a knee and hip flexion, trying to keep the trunk upright. The depth of the squatting position was individually standardized using the string barrier placed previously [56]. In order to homogenize the performances, the athletes did not receive any information regarding the horizontal displacement of the knee in the execution of the test, beyond keeping the whole foot in contact with the ground, the arms crossed on the chest and the trunk as straight as possible.

#### 2.2.2. ACL Injury Prevention Protocol (ACL-IPP)

Only participants previously listed as moderate or severe DKV were included in this protocol (*n* = 10; age: 12.68 ± 0.86 years; weight: 45.57 ± 7.44 kg; height: 157.83 ± 7.14 cm; BMI: 18.31 ± 2.43 kg/m^2^). With the objective of analyzing the acute effect that neuromuscular and proprioceptive exercises focused on the hip abductors could have on the DKV of the knee, an ACL-IPP developed specifically for this study was carried out. The exercises were always performed in the same order, with a single series of each exercise, with a one-minute recovery between exercises. Ten repetitions of the knee band squat exercise, 10 repetitions for each side of the side-steps exercise and 5 repetitions each leg in the Bulgarian split squat exercise were performed.

##### Knee-Band Squat Exercise

To standardize the squatting depth, each athlete was previously asked to perform a squat slowly, until he reached a knee angle of 90°, measured by the digital goniometer. Taking that measurement as a reference, a bench was placed at this height and they had to touch the bench in each repetition. An elastic band was placed around the knees of the participant, who had to perform the squatting exercise keeping the hip, knee and ankle aligned, preventing the elastic band from pulling the knees inward.

##### Side-Steps Exercise

With a rubber band around the knees and in a standing position and the knees semi-flexed, the participants performed lateral displacements, causing tension in the knee against the movement. The participants had to keep their hips, knees and ankles aligned, preventing the elastic band from pulling the knees inward.

##### Bulgarian Split-Squat Exercise

The starting posture was a one-leg standing position with this leg on the floor and the other supported behind, on a bench at a previously defined height by the length of the participant’s tibia (e.g., distance between the lateral malleolus and the external femoral condyle). From that position—and with an elastic band around the knee of the supporting leg—the participant had to perform the movement up to a knee flexion of 90°, measured by the digital goniometer, avoiding the displacement of the knee inwards produced by the band.

#### 2.2.3. Soccer-Specific Fatigue Protocol (SSFP)

**Figure 1:**
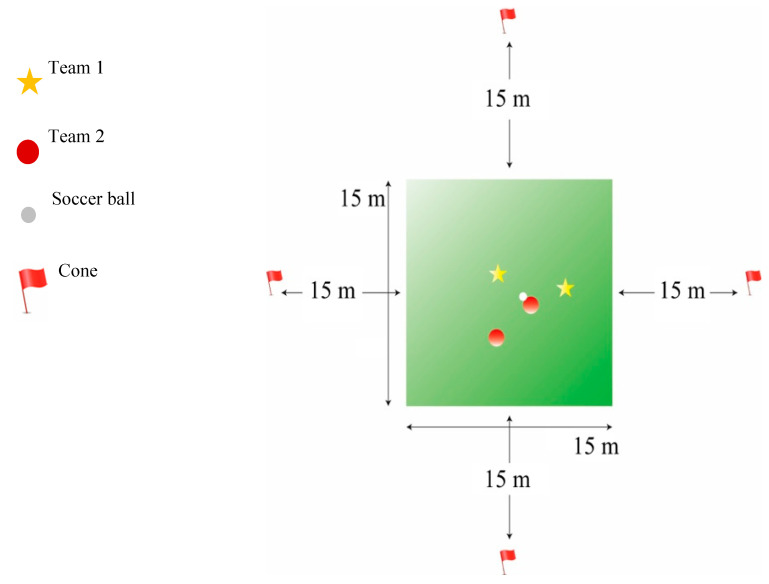
Soccer-specific fatigue protocol (SSFP) diagram.

Only participants previously categorized as light or no DKV were included in this protocol (*n* = 8; age: 12.73 ± 0.95 years; weight: 54.40 ± 13.25 kg; height: 164.04 ± 9.47 cm; BMI: 19.86 ± 2.55 kg/m^2^). To analyze whether fatigue could increase levels of DKV, a soccer-specific fatigue protocol developed explicitly for this study was carried out. The protocol consisted of a ball possession between two teams formed by two players each one, in a limited area of 15 × 15 m. One team had to keep possession of the ball, while the other had to avoid it. Every minute and by means of a whistle, all the players left the ball and performed a sprint up to a certain previously established point (with a cone), located 15 m away from the playing area. Then, they continued with the ball possession, following this procedure uninterruptedly until each individual athlete reached a fatigue level between 9–10 in the CR 0–10 scale. Figure 1 shows an example diagram of this protocol.

### 2.3. Statistical Analysis

The descriptive data of the study (age, weight, height and BMI) are shown as the mean ± standard deviation. The normality of the sample was checked by the Shapiro–Wilk test. Since the assumption of normality was not met in all variables, Wilcoxon test was used to check for differences. The effect size (ES) was calculated by the Hedges’ g, by means of the formula: $g=\frac{M_{1}-M_{2}}{SD^{*}}$, where SD* is the pooled and weighted standard deviation. Due to the small sample size, the Hedges equation was corrected and multiplied by $\left[\left(\frac{N-3}{N-2.25}\right)\sqrt{\frac{N-2}{N}}\right]$. Pre–post protocols differences (Δ) in each protocol and differentiated by leg dominance, were calculated in percentage values. Spearman correlation coefficients were calculated to analyze the relationships between age/anthropometric variables and all performance variables in the tests and protocols performed. All the analyses were performed using SPSS, v.25 (IBM Corp., Armonk, N.Y., USA). A value of *p* < 0.05 was established to determine statistical significance. Post hoc power analysis was conducted where significant differences were found between interaction effects [57].

## 3. Results

Table 1 shows the average pre–post intervention values of the two protocols performed (ACL-IPP and SSFP), differentiated by leg dominance. No statistically significant differences were found between dominant (DL) and non-dominant leg (NDL) in the pre and post-ACL-IPP assessments (*p* = 0.260, *p* = 0.721, respectively). No statistically significant differences between dominant and non-dominant leg were found in the post-SSFP assessments (*p* = 0.674), although they were found in the pre-SSFP assessments (*p* = 0.028).

**Figure 2:**
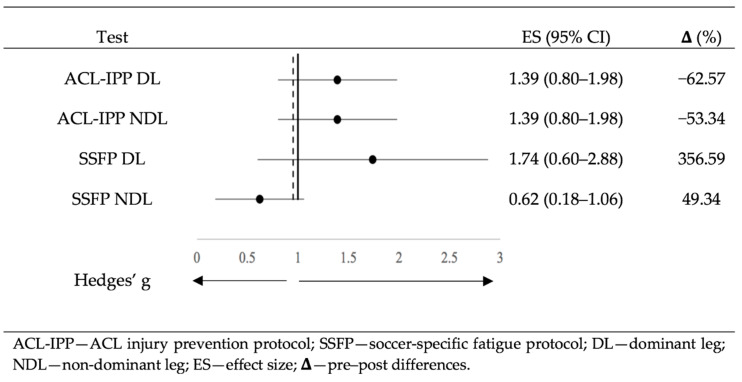
Effect sizes of protocols differentiated by leg dominance.

Figure 2 shows the effect sizes (ES) of the two protocols (ACL-IPP and SSFP) differentiated by leg dominance, as well as the pre–post differences (Δ) in percentage. According to Rhea [58], the following criteria of the effect size interpretation were followed: g < 0.25 as trivial; 0.25 < g < 0.50 as small; 0.50 < g < 1.0 as moderate; and g > 1.0 as large. The values obtained in the post hoc power analysis were: 0.992 to ACL-IPP DL and NDL, 0.997 to SSFP DL and 0.475 to SSFP NDL.

Table 2 and Table 3 show the correlations between age/anthropometric variables and pre–post-ACL-IPP and SSFP, respectively, both in DL and NDL.

**Figure 3:**
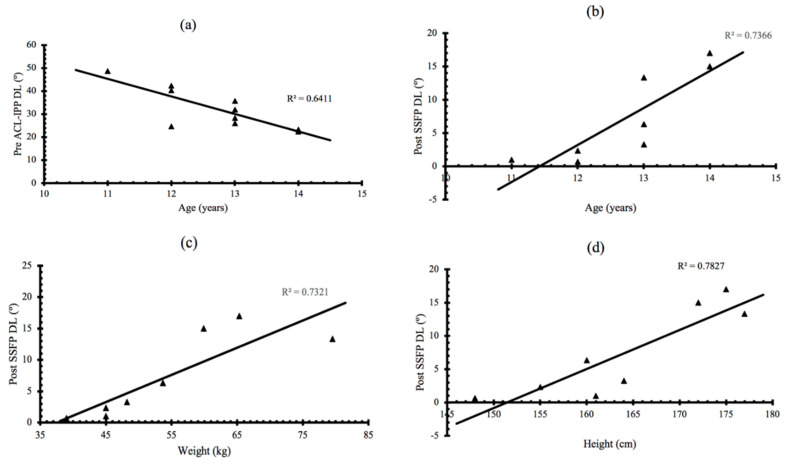
Significant correlations found between: (**a**) pre-ACL-IPP DL and age; (**b**) post-SSFP DL and age; (**c**) post-SSFP DL and weight; (**d**) post-SSFP DL and height. ACL-IPP—ACL injury prevention protocol; SSFP—soccer-specific fatigue protocol; DL—dominant leg.

Figure 3 shows the statistically significant correlations found between: (a) age and pre-ACL-IPP DL; (b) age and post-SSFP DL; (c) weight and post-SSFP DL; (d) height and post-SSFP DL. In addition, a significant correlation was found between BMI and post-SSFP DL. No statistically significant correlations were found between other variables related to age/anthropometric variables and ACL-IPP/SSFP (*p* > 0.05).

## 4. Discussion

ACL injury prevention is especially important in soccer, where many players fear ACL tears [9,12,13,14], its complications and injury recurrence [15,16,17,18,19]. One of the most important findings of the present study is that ACL-IPP significantly decreases the DKV similarly on both legs during the SLS test performance (62.57% and 53.34%, in dominant and non-dominant leg, respectively). This finding could be a contributing factor for decreasing the risk of ACL injury in sports related to landings and sudden changes of direction [2,3,24,25,26].

To date, the literature has only shown results from long-term ACL injury prevention programs in youth athletes, which have lasted between 4–10 weeks. These have reported from 18% to 67% reductions in DKV in youth male and female players of different ball sports [37,47,59,60,61,62,63,64,65]. However, the current study is based on an acute intervention as part of the specific warm-up. This makes our results highly relevant in practice, since using the ACL-IPP as part of the warm-up would be able to significantly decrease the risk of ACL injury in training or competition in the short term. This does not mean that a longer-term injury prevention program should be discontinued, but rather that the two could be perfectly compatible, with the advantages of both short-term and long-term prevention.

On the other hand, several studies have shown a 23.24% to 389.47% increase in DKV following different fatigue protocols [66,67,68,69,70,71,72]. These variable results seem to be due to the great heterogeneity of the fatigue protocols, as well as the DKV evaluation technique [73]. In reference to this heterogeneity, it was suggested that the level of fatigue [70,74] and the specificity of the fatigue exercise [75] can influence the kinematics and DKV. That is why our SSFP was designed for being as specific, intense and similar to real competition situations as possible, increasing DKV in both the DL and NDL (356.59% and 49.34%, respectively). Remarkably, it should be indicated that these increases were obtained in participants who did not have a small DKV at rest. Thus, the DKV presented by the athlete after the SSFP, could probably be very similar to the presented in a competition match, which seems to be far from the value in non-fatigue situations. Therefore, it seems that the assessment of DKV in male youth soccer players should not only be carried out in a non-fatigue situation, but also in fatigue situations [68]. This would provide a more accurate understanding of the player’s actual risk of suffering an ACL injury, which would be of great practical relevance in the area of injury prevention.

The greatest increase in the DL compared to NDL may be due to the type of activity-specific fatigue protocol applied. Since the SSFP is intended to simulate real competition, it is likely that participants will tend to use their DL to a greater extent, causing increased fatigue in this limb. This selective or localized fatigue is unlikely to occur with nonspecific soccer fatigue protocols. This may support the results of Daneshjoo and Mohseni [76], in which they also observed an increased DKV in the DL following a soccer-specific fatigue protocol in youth male. Therefore, it would probably be advisable to work on the improvement and prevention of DKV unilaterally and independently [77]. Since it seems that the values of DKV differ between both legs, it would be suggested that the dynamics of each leg should be considered individually in male youth soccer players.

In addition, our results seem to indicate an inverse significant correlation between the DKV presented in the dominant leg before the ACL-IPP and age. This may suggest an increased risk of injury at early ages, which has also been previously suggested [78]. Our study has also found direct correlations between weight, height and BMI with DKV in the dominant leg after fatigue. This may suggest that lower height and weight at early ages may reduce the risk of ACL injury [79]. This is probably not comparable to other age ranges, since the increase in muscle mass as maturation progresses causes body composition to vary [80].

To the authors’ knowledge, this is the first study focusing into the analysis of the acute effects of an ACL injury prevention program through a specific warm-up of the hip abductor muscles to reduce the DKV in male youth soccer players. Although our data are quite promising, it should be noted that our sample size was limited. However, our study was not performed with an a priori power analysis and was likely underpowered. It is proposed that future research will be able to confirm and reinforce our results using a larger sample size and an a priori power analysis, as well as analyze whether the ACL-IPP could have long-term effects. It is also suggested that future lines of research try to elucidate whether these benefits are equally applicable in the female gender and/or in other age groups. It is finally suggested that future research should examine whether the joint implementation of the ACL-IPP as part of a specific warm-up and a long-term injury prevention program may achieve better results than both performed separately. This would help to extend the range of practical application of ACL injury prevention programs, including ideally a combination of short- and long-term approaches.

## 5. Conclusions

The use of an ACL injury prevention program (based on hip abductor and external rotator neuromuscular training) as part of a soccer-specific warm-up appears to acutely reduce DKV in male youth soccer players with increased baseline DKV values during a single-leg squat. The use of a soccer-specific fatigue protocol resulted in larger baseline DKV values (especially in the dominant leg) and further validation studies may help to establish it as a protocol to detect players that require additional neuromuscular training for the prevention of DKV during game scenarios. Therefore, detection and appropriate prevention of DKV through sport-specific exercise may hold promise as a means of preventing knee injuries in male youth soccer players.

## Figures and Tables

**Table 1:** Average pre–post intervention data in the protocols differentiated by leg dominance.

Test	*n*	pre (°) (Mean ± SD)	post (°) (Mean ± SD)	95% CI		*p*-Value
				LL	UL	
ACL-IPP DL	10	32.67 ± 9.39	12.23 ± 13.91	11.04	29.82	0.007 **
ACL-IPP NDL	10	28.93 ± 7.04	13.50 ± 10.53	8.36	22.50	0.005 **
SSFP DL	8	3.11 ± 1.93	14.20 ± 6.52	6.15	16.03	0.012 *
SSFP NDL	8	8.37 ± 3.71	12.50 ± 6.00	1.18	9.43	0.123

Note: CI—confidence interval; LL—lower limit; UL—upper limit; ACL-IPP—ACL injury prevention protocol; SSFP—soccer-specific fatigue protocol; DL—dominant leg; NDL—non-dominant leg. * *p* < 0.05; ** *p* < 0.01.

**Table 2:** Correlations among age/anthropometric variables and ACL-IPP in DL and NDL.

Variables		Age	Weight	Height	BMI	pre-ACL-IPP DL	pre-ACL-IPP NDL	post-ACL-IPP DL	post-ACL-IPP NDL
Age	r	1	0.617	0.845 **	0.286	−0.778 **	0.029	−0.267	0.465
	*p*	–	0.058	0.002	0.423	0.008	0.937	0.456	0.175
Weight	r		1	0.462	0.903 **	−0.419	0.043	−0.139	0.225
	*p*		–	0.179	0.000	0.228	0.907	0.701	0.532
Height	r			1	0.195	−0.546	0.116	−0.140	0.332
	*p*			–	0.590	0.103	0.750	0.700	0.348
BMI	r				1	−0.158	−0.103	−0.224	−0.158
	*p*				–	0.663	0.776	0.533	0.663
pre-ACL-IPP DL	r					1	0.049	0.395	−0.457
	*p*					–	0.894	0.258	0.184
pre-ACL-IPP NDL	r						1	0.796 **	0.470
	*p*						–	0.006	0.171
post-ACL-IPP DL	r							1	0.383
	*p*							–	0.275
post-ACL-IPP NDL	r								1
	*p*								–

Note: BMI—body mass index; ACL-IPP—ACL injury prevention protocol; DL—dominant leg; NDL—non-dominant leg. ** *p* < 0.01.

**Table 3:** Correlations among age/anthropometric variables and SSFP in DL and NDL.

Variables		Age	Weight	Height	BMI	pre-SSFP DL	pre-SSFP NDL	post-SSFP DL	post-SSFP NDL
Age	r	1	0.789 *	0.667	0.717 *	0.652	−0.049	0.927 **	−0.210
	*p*	–	0.2	0.071	0.046	0.079	0.907	0.001	0.618
Weight	r		1	0.910 **	0.898 **	0.476	0.252	0.922 **	0.252
	*p*		–	0.002	0.002	0.233	0.548	0.001	0.548
Height	r			1	0.667	0.359	0.452	0.810 *	0.333
	*p*			–	0.071	0.382	0.26	0.015	0.42
BMI	r				1	0.407	0.095	0.833 *	0.167
	*p*				–	0.317	0.823	0.01	0.693
pre-SSFP DL	r					1	0.275	0.587	−0.156
	*p*					–	0.509	0.126	0.713
pre-SSFP NDL	r						1	0.095	0.667
	*p*						–	0.823	0.071
post-SSFP DL	r							1	0.048
	*p*							–	0.911
post-SSFP NDL	r								1
	*p*								–

BMI—body mass index; SSFP—soccer-specific fatigue protocol; DL—dominant leg; NDL—non-dominant leg. * *p* < 0.05; ** *p* < 0.01.
